# Chromosome‐dependent aneuploid formation in Spo11‐less meiosis

**DOI:** 10.1111/gtc.12998

**Published:** 2023-01-05

**Authors:** Yuri Kawashima, Arisa H. Oda, Yasushi Hikida, Kunihiro Ohta

**Affiliations:** ^1^ Department of Life Sciences, Graduate School of Arts and Sciences The University of Tokyo Tokyo Japan; ^2^ Universal Biology Institute The University of Tokyo Tokyo Japan

**Keywords:** anduploidy, aneuploid, budding yeast, meiosis, *Saccharomyces cerevisiae*, Spo11, whole genome sequencing, yeast

## Abstract

Deficiency in meiotic recombination leads to aberrant chromosome disjunction during meiosis, often resulting in the lethality of gametes or genetic disorders due to aneuploidy formation. Budding yeasts lacking Spo11, which is essential for initiation of meiotic recombination, produce many inviable spores in meiosis, while very rarely all sets of 16 chromosomes are coincidentally assorted into gametes to form viable spores. We induced meiosis in a *spo11*∆ diploid, in which homolog pairs can be distinguished by single nucleotide polymorphisms and determined whole‐genome sequences of their exceptionally viable spores. We detected no homologous recombination in the viable spores of *spo11∆* diploid. Point mutations were fewer in *spo11*∆ than in wild‐type. We observed *spo11*∆ viable spores carrying a complete diploid set of homolog pairs or haploid spores with a complete haploid set of homologs but with aneuploidy in some chromosomes. In the latter, we found the chromosome‐dependence in the aneuploid incidence, which was positively and negatively influenced by the chromosome length and the impact of dosage‐sensitive genes, respectively. Selection of aneuploidy during meiosis II or mitosis after spore germination was also chromosome dependent. These results suggest a pathway by which specific chromosomes are more prone to cause aneuploidy, as observed in Down syndrome.

## Graphical abstract

Whole genome sequencing of budding yeast *spo11*∆ spores revealed that they did not induce meiotic recombination, but frequently had unstable aneuploidy in some specific chromosomes. Our results suggest a mechanism of aneuploidy formation in specific chromosomes and provide new insights into the features of meiotic chromosome segregation.

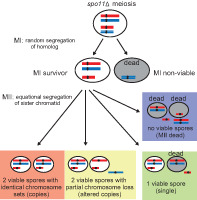

## 1 INTRODUCTION

Aneuploidy, an alteration of chromosomal makeup, interferes with the cell growth and development of an organism and is also closely related to cancer development and tumorigenesis. Aneuploidy can occur both in mitosis and meiosis by several mechanisms including chromosomal non‐disjunction. Meiosis‐derived aneuploidy has a severe impact on the reproduction of progeny by causing infertility, developmental failure, and genetic diseases. This is because meiotic recombination plays a central role in chromosome disjunction during meiosis I by forming chiasmata, which provide the physical connections between maternal and paternal homologous chromosomes (homologs), along with the formation of synaptonemal complexes and unidirectional centromere configurations. In humans, meiotic aneuploidy is not an extremely exceptional event, as previous studies have estimated that aneuploidy occurs in 2% of sperms and 20%–25% of eggs (Hassold & Patricia, 2001). Since the meiotic division of human oocytes arrests at the meiotic prophase I for many years, aneuploid is particularly prone to occur in human oocytes due to the non‐disjunction of homologs. Problematic meiotic recombination causes the homolog non‐disjunction in meiosis I and induces aneuploid formation in human eggs or sperms, which leads to genetic diseases such as Down syndrome (Hassold et al., 2007; Lejeune et al., 1959) or Klinefelter syndrome (Philip et al., 1976; Thomas & Hassold, 2003). The relationship between impaired meiotic recombination and aneuploidy is observed in many other eukaryotes, not only in humans. Failure or loss of meiotic recombination universally results in infertility or aneuploid formation in many eukaryotes.

Initiation of meiotic recombination is triggered by the programmed DNA *d*ouble‐*s*trand *b*reaks (DSBs) formed by the evolutionally conserved meiosis‐specific Spo11‐Top6BL transesterase (Bergerat et al., 1997; Keeney, 2008; Keeney et al., 1997; Klapholz et al., 1985; Nichols et al., 1999; Robert et al., 2016; Vrielynck et al., 2016). After the cleavage, Spo11 is covalently attached to 5′‐ends of DSBs forming Spo11‐oligo complexes (Neale et al., 2005), which are resected by Mre11‐Rad50‐Xrs2/Nbs1complex, Com1/Sae2/CtIP, and the exonuclease Exo1. Spo11 can also introduce concerted double DSBs separated by 33 to more than 100 base pairs, generating double‐stranded gaps which require subsequent gap repair homologous recombination (Johnson et al., 2021; Prieler et al., 2021). DNA cleavage activity of Spo11 has been considered to be regulated at multi‐layered levels such as accessory proteins (Arora et al., 2004; Claeys Bouuaert et al., 2021; Henderson et al., 2006; Li et al., 2006; Maleki et al., 2007; Malone et al., 1991; Panizza et al., 2011; Sasanuma et al., 2007, 2008), chromatin and epigenetic modifications (Acquaviva et al., 2013; Berchowitz et al., 2009; Borde et al., 2009; Buard et al., 2009; Ohta et al., 1994, 1998; Sommermeyer et al., 2013; Székvölgyi et al., 2015; Wu & Lichten, 1994; Yamada et al., 2004), and higher chromosome structure levels (Kariyazono et al., 2019; Kugou et al., 2009; Lam & Keeney, 2015; Lange et al., 2016; Miyoshi et al., 2012; Murakami et al., 2020; Pan et al., 2011; Shodhan et al., 2022).

Spo11 contributes essentially to meiotic recombination, though meiotic defects in *spo11*∆ can be alleviated at least partially by ionizing irradiation (Dernburg et al., 1998) and artificially induced single‐ or double‐strand DNA breaks due to replication errors, mismatch repair, and base excision repair (Farah et al., 2005; Pauklin et al., 2009). The budding yeast *spo11*∆ mutant does not form DSBs and exhibits an abolishment of meiotic recombination and chiasmata formation. Spores can be formed in *spo11*∆, but those spores are almost inviable spores due to a lack of proper chromosome segregation (Klapholz et al., 1985; Kugou et al., 2007). Spo11‐dependent DSB formation is important for the promotion of the synapsis formation and the synaptonemal complex (Baudat et al., 2000; Celerin et al., 2000; Romanienko & Camerini‐Otero, 2000), except for some organisms such as *Drosophila melanogaster* and *Caenorhabditis elegans*. Given the characteristics of the DSBs introduced by Spo11 and their rigorous regulatory mechanisms, the role of Spo11 and Spo11‐dependent meiotic recombination may be more complex and multifunctional than previously thought (Bellani et al., 2010; Celerin et al., 2000; Cha et al., 2000; Hou et al., 2021; Kugou et al., 2009). However, since very few (<0.25%) viable spores are produced in *spo11∆* (Klapholz & Esposito, 1982), their genome structure has not been extensively investigated.

In this study, we isolated tetrads of a *spo11*∆ diploid of the budding yeast *Saccharomyces cerevisiae*, in which pairs of homologs derived from both parents can be distinguished by single nucleotide polymorphisms (SNPs), and analyzed whole genome sequences of viable spores. The results showed that the *spo11*∆ mutant did not induce recombination, such as gene conversion or crossover, and generated fewer point mutations. In *spo11*∆ which induces nondisjunction of achiasmatic homologs, aneuploidy occurs very frequently in some specific chromosomes, which often show instability during mitotic growth. It is known that aneuploidy preferentially occurs during meiosis in certain chromosomes and also in human genetic diseases such as Down syndrome. The present results suggest that the chromosome‐dependent incidence and stability of aneuploidy during meiotic and mitotic divisions may lead to the abnormalities of specific chromosomes.

## 2 RESULTS

### 2.1 Sporulation profiles of the wild‐type and spo11∆ strains for genome resequencing

**FIGURE 1:**
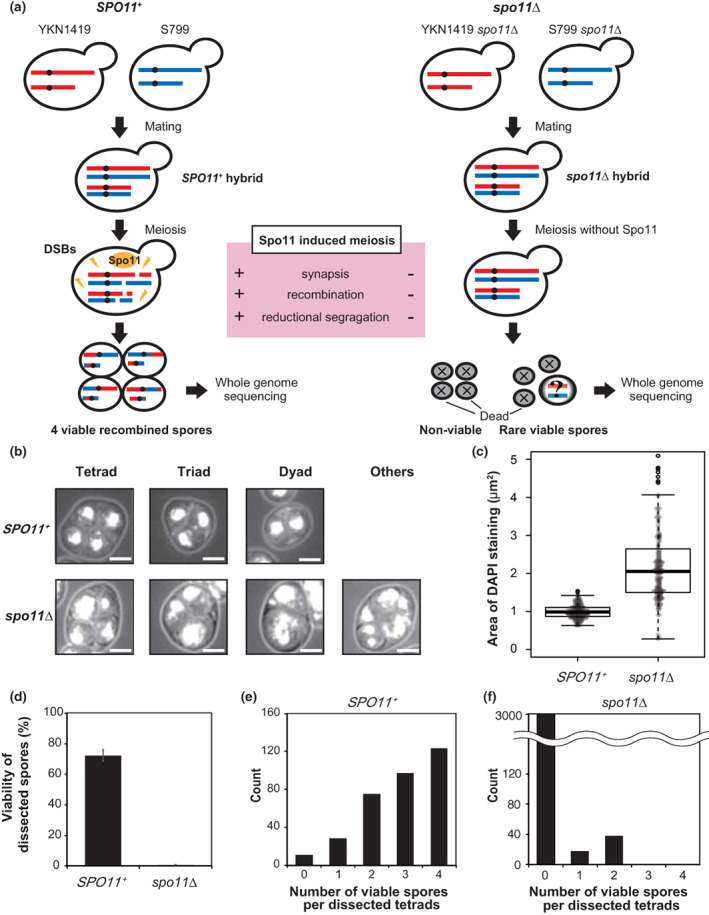
Experimental design and phenotypes of the tester wild‐type and *spo11*∆ diploids during meiosis and spore formation. (a) Schematic drawings of whole genome sequencing (WGS) using hybrid diploids with different genetic backgrounds (S799, SK1‐background, and YKN1419, S288C‐background) for *SPO11* ^+^ wild‐type and *spo11*∆. (b) Fluorescent microscopic images of DAPI stained *SPO11* ^+^ and *spo11*∆ spores 24 hours after meiotic induction. Asci harboring tetrads, triads, dyads, and other polyads are shown. The scale bar indicates 2.5 μm. (c) Distribution of DAPI‐stained area for nuclear DNA in *SPO11* ^+^ and *spo11*∆ spores. The t‐test showed a significant difference (*p* = 4.04 × 10^−13^; *SPO11* ^+^, *n* = 148, standard deviation (SD) = 0.017; *spo11*∆, *n* = 160, SD = 0.78). (d) Spore viability of dissected tetrads. Exactly 334 *SPO11* ^+^ tetrads and 3137 *spo11*∆ tetrads were dissected and germinated on nutritious medium (YPD) plates at 30°C to examine their viability (three biological replicates, mean + SD). (e,f) Distribution of viable spore numbers in a dissected tetrad of (e) *SPO11* ^+^, (f) *spo11*∆ in (d).

To distinguish the parental homologs and detect recombination events extensively, hybrid strains were generated by mating two parental strains YKN1419 (S288c background) and S799 (SK1 background) with 0.7% SNPs distributed across the entire genome (Figure 1a), as described in the previous studies (Anderson et al., 2011; Mancera et al., 2011; Muramoto et al., 2018; Qi et al., 2009).

The flow cytometry analyses revealed that the transition to meiosis I and meiosis II began slightly earlier in *spo11*∆ diploids than in the wild‐type (Figure S1a,b), showing that the S phase length was shortened by about 25% in *spo11*∆ as reported previously (Cha et al., 2000). Early meiotic budding yeast cells can return to the mitotic division cycle after transferring meiotic cells to a nutrient‐rich medium. This phenomenon is exploited in the *R*eturn *t*o *G*rowth (RTG) experiment (Esposito & Esposito, 1974), which allows us easier monitoring of the early meiotic process without analyzing dissected spores. In the RTG experiments, the survival rate of the *spo11*∆ strain decreased over time to <20%, whereas the survival rate of the wild‐type strain remained at higher levels (about 60%) (Figure S1c), suggesting that the *spo11*∆ cells died during meiotic segregation.

Budding yeast diploids normally produce tetrads (four spores) in one ascus after the completion of meiosis. The spore formation rate of the wild‐type and *spo11*∆ was 97.0% and 91.5%, respectively (Figure S1d), which is consistent with the previous observation using a similar hybrid strain (88%) (Laureau et al., 2016). We observed DAPI‐stained spores of wild‐type and *spo11*∆ strains to determine how many nuclei were separated within one ascus. Although the number of spores within each asci is distributed at the same proportion as in the wild‐type (Figure S1e), microscopic observation revealed that *spo11*∆ often produced larger uneven‐sized nuclear DNA clumps (Figure 1b,c). Tetrad analysis of the wild‐type and *spo11*∆ asci indicated 72.4% and 0.76% (91/12,548 dissected spores in 3137 asci) of dissected spores were viable, respectively (Figure 1d), comparable to the previous observation (wild‐type 71%, Laureau et al., 2016; *spo11*∆, 0.25%, Diaz et al., 2002). We found that 37% of the wild‐type asci had four viable spores, whereas no *spo11*∆ asci included four viable spores at all (Figure 1e,f).

### 2.2 Genomic resequencing of viable spores of spo11∆

We then conducted genomic resequencing of viable spores of the wild‐type (72 viable spores in 18 dissected tetrads harboring four viable spores) and *spo11∆* (91 viable spores from 3137 dissected tetrads). We could obtain complete genomic sequences of spores from the wild‐type (4 × 18 = 72 spores) and *spo11∆* (69 spores: 1 viable spore in one ascus, 17 cases, 1 × 17 = 17 spores; 2 viable spores in one ascus, 26 cases, 2 × 26 = 52 spores) (Figure S2). From the sequencing results, we observed marked differences in recombination events (Table 1). Using the RecombineX tool, we detected 78.5 crossing overs (COs) per tetrad and 110 gene conversions (GCs) per tetrad in the wild‐type strain as reported previously (Li et al., 2022), whereas *spo11*∆ cells did not induce any types of recombination. Therefore, nondisjunction of achiasmatic homologs is expected to occur in *spo11*∆. The occurrence of single nucleotide variations (SNVs) in the wild‐type strain during meiosis was four times as much as that in the *spo11*∆ strain as described (Mansour et al., 2020; Rattray et al., 2015; Simchen et al., 2021). Positions of Spo11‐independent SNVs showed seemingly little correlation with meiotic DSB hotspots considering the average interval (2.2 kb) between hotspots (Pan et al., 2011; Table 1).

**TABLE 1:** Summary of whole genome analysis

	*SPO11* ^+^	*spo11∆*
Crossing over (CO)	78.5	0
Gene conversion (GC)	109.8	0
GC/CO	1.4	—
Aneuploid*	0/72	66/69
SNV events	8	2
1.0 kb ≤ DSB	4	1
0.5 kb ≤ DSB <1.0 kb	1	0
0.2 kb ≤ DSB <0.5 kb	2	0
DSB <0.2 kb	1	1

*Note*: WGS was performed as described in Figure 2. We counted events of crossing overs (COs), gene conversions (GCs), aneuploid formation, and meiotically induced single nucleotide variations (SNVs). Average frequencies of COs and GCs per tetrad are indicated in parenthesis. SNV events were further classified by the distance from the nearest meiotic DSB hotspots (Pan et al., 2011). Distances from the hotspots were classified into four categories: over 1.0 kb, 0.5–1.0 kb, 0.2–0.5 kb, and under 0.2 kb.

* The ploidy of each strain determined by NGS analysis.

We then estimated the ploidy variation of viable spores. The wild‐type four viable spores were all haploid euploids (Table 1), while viable spores of *spo11*∆ exhibited several types of ploidies. For instance, three spores harbored a complete diploid set of all chromosomes (diploid euploids) and the other 66 spores had a complete haploid set of all chromosomes but with aneuploidy (Table 2).

**TABLE 2:** Summary of classification of *spo11*∆ viable spores

Types of viable spores in *spo11*∆ cases	Number of strains (in 69 strains)
1 viable spore in a tetrad diploid with the complete homolog set aneuploid	1
	16
2 viable spores in a tetrad: identical copy diploid with the complete homolog set aneuploid	2
	38
2 viable spores in a tetrad: non‐identical copy aneuploid	12

*Note*: Ploidy status of *spo11*∆ viable spores. In the case of a single viable spore in one ascus, we observed one spore with the complete diploid set of all chromosomes or 16 viable spores having all chromosomes but with aneuploidies. In the case of two viable spores in one ascus, one pair of viable spores contained the complete diploid set of all chromosomes, and the other 38 spore pairs had all chromosomes but with identical copies of aneuploidy. We also detected 12 cases of two viable spores in one ascus containing non‐identical copies of aneuploid chromosomes.

**FIGURE 2:**
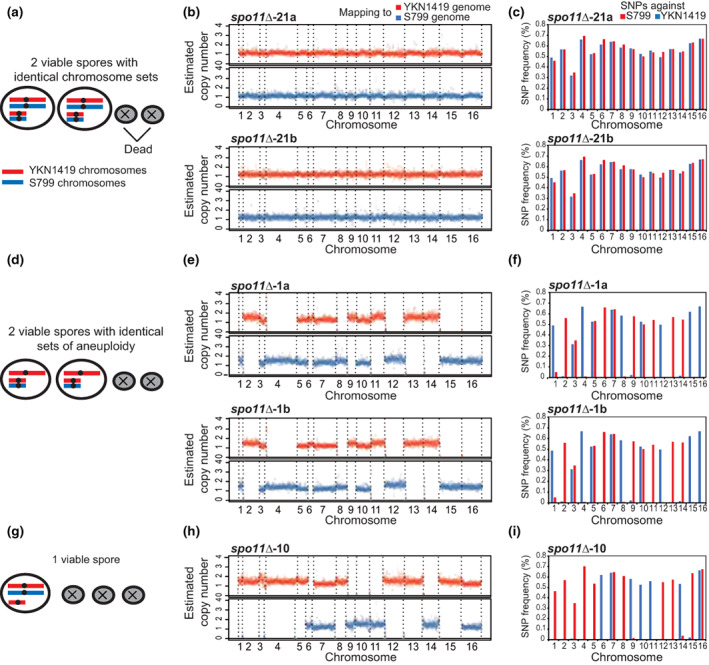
Aneuploid formation in *spo11*∆ viable spores revealed by WGS analyses. (a) A schematic drawing of two viable spores in one ascus with complete diploid chromosome sets. (b) Sequence read mappings of the case of (a) (an example of *spo11*∆*‐21a* and *spo11*∆*‐21b* spore‐derived strains). Both strains have a complete diploid set of chromosomes. (c) SNP frequency plot of each chromosome of *spo11*∆*‐21a* and *spo11∆‐21b*. SNP frequencies against YKN1419 and S799 genomes exhibited almost the same levels. (d) A schematic drawing of two viable spores in one ascus with identical patterns of aneuploidy. (e) Sequence read mappings of the case of (d) (*spo11∆‐1a* and *spo11*∆*‐1b* spore‐derived strains). Note that mapping patterns were almost identical. In addition, chromosomes 3, 5, 7, and 10 showed mapping against both YKN1419 and S799 genomes, while other chromosomes exhibited mappings against either genomes. (f) SNP frequency plot of each chromosome of *spo11*∆*‐1a* and *spo11*∆*‐1b*. In both strains, chromosome 3, 5, 7, and 10 formed disomy. (g) A schematic drawing of a single viable spore in one ascus. (h) Sequence read mappings of the case of (g) (*spo11*∆*‐10* spore‐derived strain). (i) SNP frequency plot of each chromosome of *spo11*∆*‐10*. Chromosome 7 and 16 formed disomy.

Next, using the SNP information between YKN1419 (S288c background) and S799 (SK1 background), homologs of the viable *spo11∆* spores were determined which parental strain they were derived from (Figure S3a–d). We numbered the *spo11*∆ tetrads containing viable spores and assigned “a” and “b” if 2 viable spores were obtained from that tetrad (e.g., *spo11*∆‐21a, *spo11*∆‐21b). Figure 2a–c represent the results of 2 viable spores in one ascus with a complete diploid set of all chromosomes (*spo11*∆‐21a and *spo11*∆‐21b) and indicated those spores had each set of maternal and paternal homologs (diploid euploids). Therefore, these diploid euploid spores were likely generated by accidentally bypassing reductional segregation in meiosis I, followed by equational segregation of sister chromatids in meiosis II, as observed in *spo13* mutants which makes diploid dyad (Klapholz & Esposito, 1980). Spo13 is meiosis I specific protein which prevent sister chromatid segregation and mediate kinetochore mono orientation (Galander et al., 2019).

Figure 2d–f demonstrates the results of 2 viable spores in one ascus carrying a complete haploid set of all chromosomes with identical patterns of aneuploidy (*spo11*∆‐1a and *spo11*∆‐1b). In this case, chromosomes 3, 5, 7, and 10 exhibited disomy. The occurrence of two spores with identical patterns of aneuploidy in one ascus suggests that aneuploidy was generated during meiosis I. Another example was only one viable spore in one ascus with disomy of chromosomes 7 and 16 (*spo11*∆‐10, Figure 2g–i). We did not observe any trisomy or larger polysomy in *spo11*∆ viable spores.

**FIGURE 3:**
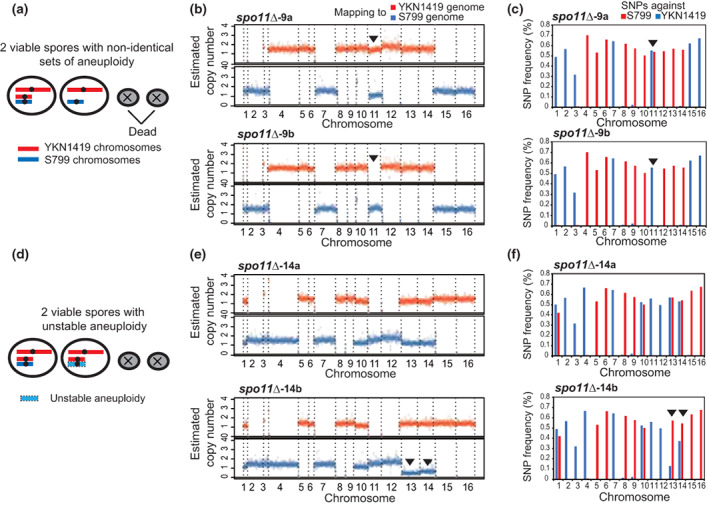
Viable spore pairs with altered ploidy profiles. (a) A schematic drawing of two viable spores with altered patterns of aneuploidy. (b) Sequence read mappings of the case of (a) (*spo11*∆‐9a and *spo11*∆‐9b spore‐derived strains). Filled arrowheads show aneuploid chromosomes with altered copy number between the spore pair. (c) SNP frequency plots of each chromosome of *spo11*∆‐9a and *spo11*∆‐9b. Although almost all chromosomes showed identical patterns in both strains, chromosome 11 exists only in *spo11*∆‐9a. Filled arrowheads show aneuploid chromosomes with altered copy number between the spore pair. (d) A schematic drawing of two viable spores where copy number of some aneuploid chromosomes are partially reduced. (e) Sequence read mappings of the case of (d) (*spo11*∆‐14a and *spo11*∆‐14b spore‐derived strains). Filled arrowheads show aneuploid chromosomes with reduced copy numbers. (f) SNP frequency plots of each chromosome of *spo11*∆‐14a and *spo11*∆‐14b. In *spo11*∆‐14a, chromosomes 13 and 14 exhibited partial reduction in copy number. Filled arrowheads show aneuploid chromosomes with reduced copy numbers.

Notably, we observed a few *spo11*∆ asci with two viable spores in which only a few chromosomes differ, although the aneuploid pattern is almost identical (Figure 3a–c, see chromosome 11 in *spo11*∆‐9a and *spo11*∆‐9b). In addition, we found cases of two viable spores in one ascus with the same aneuploid pattern, but with the partial copy number reduction of specific chromosomes (Figure 3d–f, see chromosomes 13 and 14 in *spo11*∆‐14b; and S4 a‐b). This observation suggests that some aneuploids are not stable and easy to be lost during mitotic divisions after the spore germination. Thus, we next determined the incidence of aneuploids by utilizing the thresholds calculated from SNP frequency for ploidy determination (Figure S3e,f).

### 2.3 The aneuploidy incidence depends on chromosomes

**FIGURE 4:**
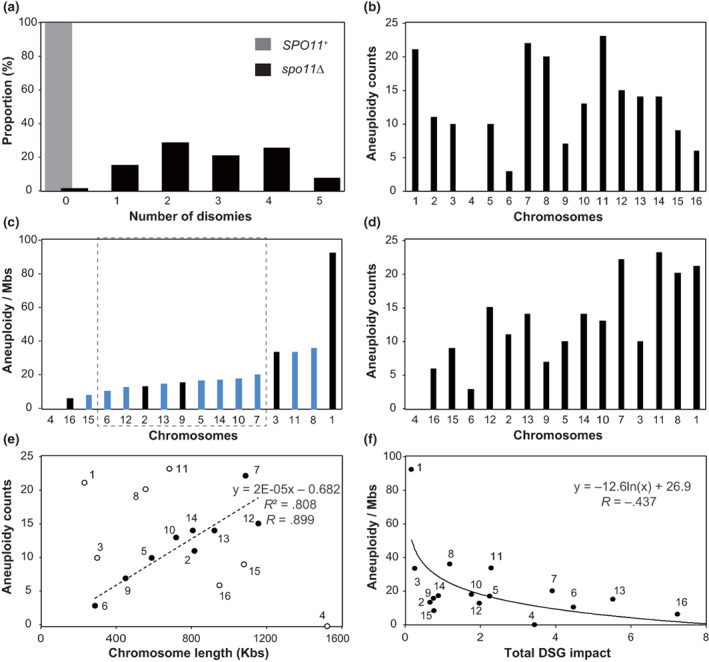
Aneuploidy frequencies correlate with the chromosome length and the chromosomal tolerance for dosage sensitive genes (DSG). (a) Distribution of numbers of disomy in *SPO11* ^+^ and *spo11*∆ spores are shown. Average number of disomy of wild‐type and *spo11*∆ were 0.0 and 2.7, respectively. Black and gray bars represent results of *SPO11* ^+^ and *spo11∆*, respectively. (b) The aneuploidy counts of each chromosome. Horizontal axis is sorted by the chromosome number. (c) The aneuploidy counts normalized by chromosome length (counts/Mbs). The horizontal axis is arranged in ascending order of the normalized frequency. Dotted square marks a subgroup of chromosomes excluding the highest four chromosomes (over 25 counts/Mbs) and the lowest three chromosomes (below 10 counts/Mbs). Blue and gray bars represent chromosomes with unstable and stable aneuploidy, respectively. (d) The aneuploidy counts of each chromosome. The horizontal axis is arranged in ascending order of the normalized frequency as in (c). (e) Correlation between chromosome length and the aneuploidy count normalize by chromosome length. Black circles show the subgroup chromosomes in (c), and open circles show the highest four and the lowest three chromosomes. (f) Correlation between aneuploidy frequency and the total DSG impact for each chromosome. The numbers indicate the chromosome number. Black curve shows approximated log curve.

We observed at most five disomic events per strain within 66 aneuploid *spo11*∆ viable spores (Figure 4a). The frequency of disomy formation varied among chromosomes: no disomy was detected in chromosome 4, but multiple disomic events were detected in other chromosomes (Figure 4b). Previous studies reported that the rate of human aneuploid shows a negative correlation with chromosome length (Torres et al., 2008). Therefore, we calculated the total numbers of aneuploidy (disomy) formations per chromosome length and arranged these data, and demonstrated aneuploidy counts in ascending order on graphs (Figure 4c,d, respectively). Many chromosomes (chromosomes 6, 2, 12, 13, 9, 5, 14, 10, and 7, indicated within the dashed rectangle in Figure 4c) showed relatively constant values (10–20 disomy formation per mega‐base pair, Mbp), suggesting that the aneuploid formation in *spo11*‐deficient meiosis generally depends on chromosome length. Indeed, a linear approximation of aneuploid counts and chromosome lengths among these selected chromosomes (i.e., chromosomes 2, 5, 6, 7, 9, 10, 12, 13, and 14, with the dashed rectangle in Figure 4c) showed a high correlation (*R* = .899) (Figure 4e). This shows a contrast with the negative correlation of human aneuploidy formation with chromosome length. However, when we include all the chromosomes in the linear approximation, the correlation coefficient was −0.184. This is because the aneuploid incidences of several chromosomes such as chromosomes 1, 3, 4, 8, 11, 15, and 16 showed no correlation with chromosome lengths. Notably, two groups of chromosomes exhibited very low or very high aneuploid counts per chromosome length (chromosomes 4, 15, 16, or 1, 3, 8, 11, respectively). We also examined the correlation between chromosomes showing unstable aneuploidy (indicated with blue bars in Figure 4c) and the aneuploid incidence normalized with chromosome length but did not observe any apparent correlation.

We further investigated the correlation between the aneuploid formation and the dosage‐sensitive genes (DSGs), which do not allow high‐copy existence when they are placed on multicopy plasmids (Makanae et al., 2013). Since Bonney et al. reported that aneuploid proliferation defects in yeast are not explained by copy number changes of a few DSGs (Bonney et al., 2015), we assumed that multiple DSGs resided on a single chromosome act cumulatively. Then, to estimate the overall impact of the DSGs on the chromosomes, the inverse of the copy number limit for each DSG was calculated and summed for each chromosome, which was defined as the total DSG Impact. Since each total DSG impact for a chromosome (Figure S5) may affect aneuploid incidence synergistically, we conducted a logarithmic approximation of both values and found that the two values turned out to be inversely correlated at *R* = −.437 (Figure 4f).

We also examined the bias of the partitioning of homologs. In all spores analyzed in this study, the frequency of YKN1419‐derived and S799‐derived homologs was almost 50% in the wild‐type (Figure S6a,b,e), indicating an even distribution. However, in *spo11∆* viable spores, YKN1419‐derived homologs exhibited a slightly smaller frequency (Figure S6c,d,e), suggesting there is a preference for S799‐derived homologs in the meiotic segregation of *spo11*∆ cells. In addition, such preference differed among chromosomes: chromosomes 1, 3, 4, and 13 exhibited even or YKN1419‐preferred segregation patterns (Figure S6d).

### 2.4 Evaluation of the aneuploid instability

**FIGURE 5:**
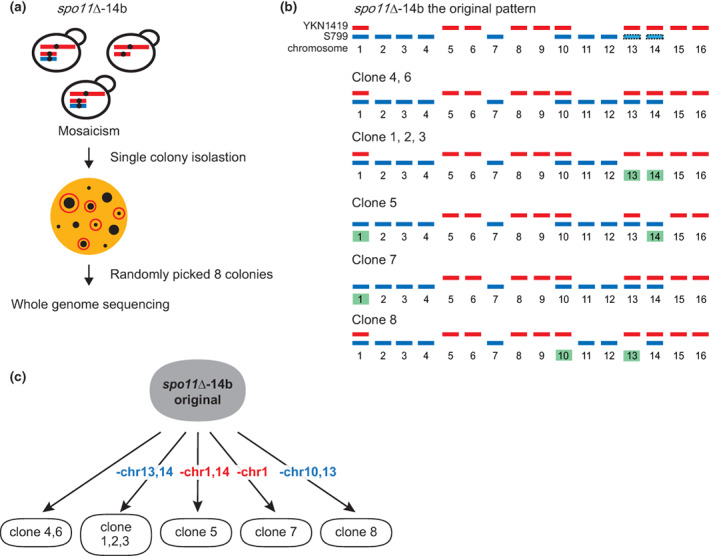
Investigation on mosaicism in the *spo11*∆‐14b progenies with aneuploidies showing altered copy number. (a) Schematic image of single colony isolation and WGS of the *spo11*∆‐14b progenies shown in Figure 3e,f. (b) Schematic demonstrations of karyotypes of 8 clonal colonies. Clone 4 and 6 were the same karyotype as original. Clone 5 and 7 lost some YKN1419‐derived chromosomes. Clone 1, 2, 3, and 8 lost some S799‐derived chromosomes. Green highlights indicate the regions of dropped chromosomes compared with the original pattern. (c) Outlines of the aneuploidy loss shown in (b).

In Figure 3, we mentioned the possibility that some aneuploids are not stable and easy to be lost during mitotic divisions after spore germination. To test this notion, we examined the stability of aneuploids during nutritional growth of *spo11∆*‐14b‐derived eight independent isolated colonies (Figure 5a). Whole genome sequencing of these colonies revealed that some disomy were preferentially eliminated during cell proliferation (Figure 5b,c, Figure S7). Chromosome 1 was lost in clone 5 and 7. Chromosomes 13 or 14 were eliminated in clone 1, 2, 3, and 8 or clone 1, 2, 3, and 5, respectively. Chromosome 10 was lost in clone 8. All four of the disomic chromosomes in *spo11*∆‐14b were lost in either of the isolated colonies.

**FIGURE 6:**
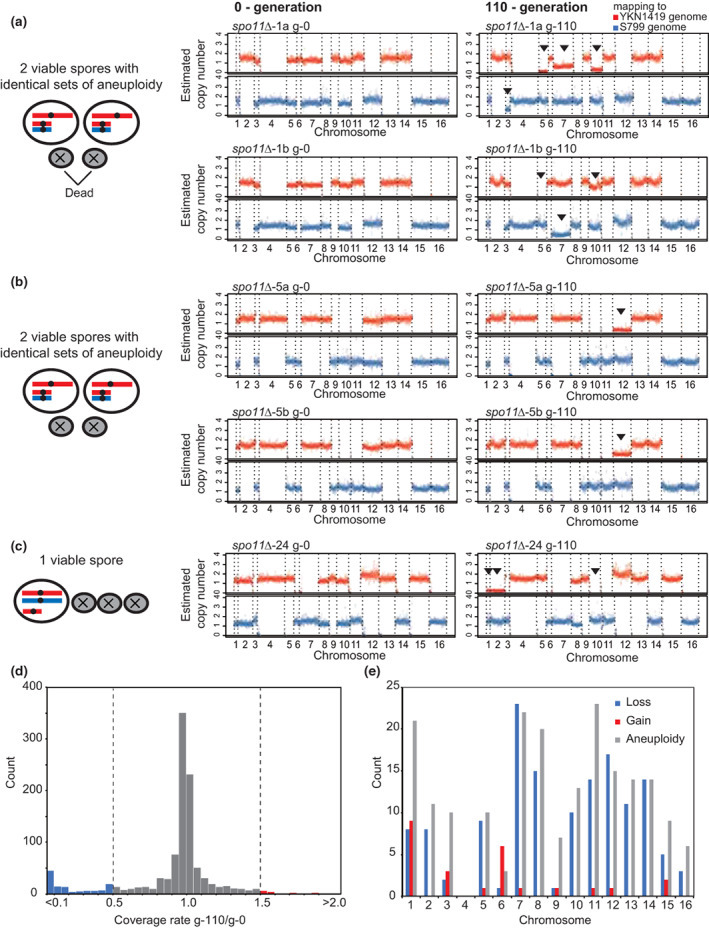
Alteration of chromosome copy number during mitotic passages. (a–c) The sequence mapping data of cases with altered aneuploid copy number at 110 generations. Middle panels show mapping profiles at g‐0 and right panels show those at g‐110. Arrowheads indicate the chromosomes with reduced aneuploid copy numbers. (a) Example of two viable spores in one ascus showing different patterns of reduced aneuploid copy number after 110 generations. (b) Example of two viable spores in one ascus showing the same patterns of reduced aneuploid copy number after 110 generations. (c) Example of one viable spore in one ascus with reduced aneuploid copy number after 110 generations. (d) Histogram of the ratio of coverages at generation 110 (g‐110) versus coverages at generation 0 (g‐0) (g‐110/g‐0) calculated for each chromosome in each strain. The cases below the lower threshold 0.5 and above the upper threshold 1.5 are marked in blue and red, respectively. (e) The count of aneuploidy with altered copy numbers of each chromosome after 110 generations. Blue bars indicate the cases with reduced copy number. Red bars indicate the cases of gained copy number. The aneuploidy frequency of each chromosome are also indicated with gray bars as shown in Figure 4b.

We further studied the aneuploid stability of all chromosomes except chromosome 4 more comprehensively: 69 of *spo11*∆ viable spores were cultured for 110 generations in a nutrient medium, and 56 strains were successfully isolated. Whole genome sequencing was conducted with these cells (g‐0, generation 0; and g‐110, generation110) and the representative examples were shown (Figure 6a–c). The ratios of g‐110 coverages to g‐0 coverages were calculated for each chromosome in each strain, and their distribution was shown in the histogram (Figure 6d). By referring to the mapping results (Figure 6a–c), we evaluated a reduction in chromosome copy number when the value of g‐110/g‐0 was <0.5 (Figure 6d, shown in blue) and a gain in chromosome copy number when the value was greater than 1.5 (Figure 6d, shown in red).

In most cases, chromosome copy numbers remained constant. Some chromosomes exhibited a reduction in their copy numbers, but the frequency of the copy number gain was much smaller (Figure 6d). We then examined and plotted the number of copy number alterations after 110 generations for each chromosome except chromosome 4 (Figure 6e). Some chromosomes, such as chromosomes 3 and 9, remained relatively stable once aneuploidy was established, while others, such as chromosomes 2, 5, 7, 8, 10, 11, 12, 13, and 14, were unstable and prone to alter their copy numbers. Cells with these unstable aneuploidies are expected to be gradually outcompeted by euploid‐type cells during multiple rounds of mitotic division. The chromosome‐dependent instability in mitosis is generally consistent with the results of previous studies using aneuploid yeast strains obtained from triploid recombination‐proficient (*SPO11* ^+^) meiosis (Zhu et al., 2012), although there are differences in the observed instability of each chromosome. For example, chromosome 7 was unstable in this study (Figure 6e), but was relatively stable in the analysis by Zhu et al. Such difference may be explained by the difference in tolerance of aneuploid which may depend on the number of extra chromosomes. We studied viable spores of recombination‐deficient (*spo11∆*) meiosis which generates fewer extra chromosomes, while Zhu et al. examined triploid meiosis‐derived viable spores with more abundant extra chromosomes.

### 2.5 Simulation of aneuploid formation

This study revealed that Spo11‐deficient meiosis often leads to the formation of two spores of identical aneuploid composition. This observation is explained by the notion that aneuploidy in Spo11‐deficient meiosis is formed in random segregation of homologs during meiosis I followed by equational segregation of sister chromatids during meiosis II. According to this model, we then tried to predict how or by which pathway aneuploid spores were generated by conducting computer simulations and comparing them with the actual data.

**FIGURE 7:**
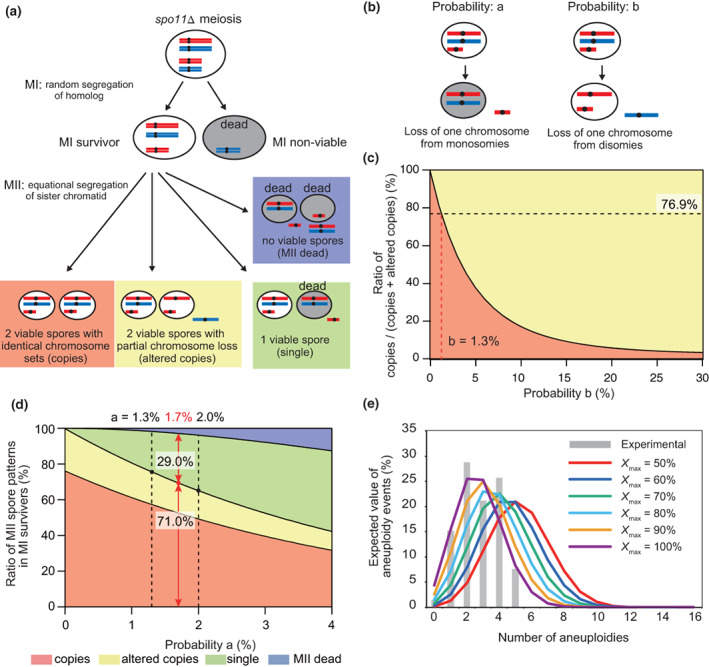
Simulation of aneuploidy formation and chromosome loss during *spo11*∆ meiosis. (a) A schematic drawing of a model for the meiotic chromosomal segregation in *spo11*∆. We assume random chromosome segregation in meiosis I (MI) and equational segregation of sister chromatids with accidental loss of some chromosomes in meiosis II (MII). Spore generated after MII can be classified into four categories according to the types of chromosome distributions. (b) The definition of probability a (Pa) and b (Pb) during MII in the above model. (c) Simulation for the proportion of two spores in one ascus with the same chromosome/aneuploid compositions (“copies”) in all types of viable two spores in one ascus, when Pb was given in the range of 0% to 30% at Pa = 0. The observed frequency was 76.9% (black dashed line) and its corresponding Pb value is 1.3% (red dashed line). (d) Proportion of four MII spore patterns in all MI survivors when Pb = 1.3%. Red, yellow, green and blue regions represent the ratio of copies, copies with altered aneuploid copy number (altered copies), single viable spore in one ascus (single), and presumed dead cells in MII (MII dead). The observed proportion of single (green) in all viable spore sets (green + yellow + red) was 71.0% ± 5.9%, which is corresponding to Pa = 1.7% (a red dashed line). Two black dashed line indicate the range of Pa value from 1.3% to 2.0% corresponding to observed range of standard error. (e) Distribution of numbers of aneuploidy in *spo11*∆ spores at various X_max_ values. Gray bars indicate the experimentally observed results. Red line (*X*_max_ = 50%) indicates non‐biased segregation simulation results at Pa = 1.7% and Pb = 1.3% as in (c) and (d). Other colored lines indicate biased simulation results at various chromosome length dependent bias for *X*_max_ = 60%–100%. Vertical axis indicates the expected value of aneuploidy events (%). Horizontal axis indicates the number of aneuploidies per one cell.

Sporulation of *spo11*∆ diploids with two sets of 16 homologs was simulated with 10^8^ spores at different probabilities of chromosome loss. The numbers of duplicated chromosomes were then checked, and their survival rates were calculated. In this simulation, we made some assumptions. First, each chromosome does not have any harmful or beneficial factors in forming aneuploidy. Second, more than one copy of all 16 chromosomes should exist in a spore to be viable. Third, *spo11∆* diploids undergo random chromosome segregation in meiosis I, and the subsequent equational segregation of sister chromatids in meiosis II (Figure 7a). Fourth, two viable spores with the same aneuploid set may lose some of their chromosomes and sometimes result in the formation of a single surviving spore (Figure 7a). We also established two parameters: One is the probability of eliminating monosomes during meiosis II (Pa), and the other is the probability of losing one of the disomic pairs (Pb) (Figure 7b). Although Pa and Pb are assumed to be nearly equivalent, they were set as different parameters in this simulation. The schematic flowchart of the simulation is described in Figure S8.

According to the assumption described above, we first estimated the ratio of two spores with a complete pair of homologs from both parents (copies) versus all types of viable two spores under various Pb (0%–30%) (Figure 7c). The experimentally observed ratio was 76.9% (20 pairs of copies versus 26 pairs of all types of viable two spores), which was corresponding to the Pb = 1.3% (indicated with dashed lines in Figure 7c). We then used this value (Pb = 1.3%) and estimated the frequencies of copies, altered copies, single viable spores (single), and dead cells (MII dead) under various Pa values (0%–4%) (Figure 7d). Since the value is consistent with the observed rate of 71.0% (the rate of all types of two viable spores vs. all types of viable spores), we estimated Pa to be 1.7% according to the single spore proportion (indicated with red arrows and black dashed lines of standard error in Figure 7d). This Pa value is comparable with the estimated Pb value (1.3%).

Then we incorporated these values (*a* = 1.7%, *b* = 1.3%) into the simulation and demonstrated the distribution of aneuploid events in a single spore. The results showed that five aneuploidy events (disomy) were most often found in a single spore. However, the observed frequent number of disomy per spore was between two to four (Figure S9), which is smaller than the simulated results. This discrepancy may be due to the simulation assumption that each chromosome does not have any harmful or beneficial factors in forming aneuploidy, but each chromosome has different lengths and numbers of DSGs which have positive and negative impacts on the aneuploidy formation of the chromosomes, respectively. The segregation of homologs during MI might be influenced by chiasma‐independent homolog pairing observed in early meiosis (Weiner & Nancy, 1994) which is assumed to strengthen in proportion with chromosome length. We indeed observed linear correlation between the aneuploid counts and chromosome length (Figure 4e).

We, therefore, tried a refined simulation considering chromosome length‐dependency by adding an additional parameter: the MI segregation probability of each homolog depending on the chromosome length. Average length of homologous chromosome *N* (*N* = 1, 2, …, 16) of YKN1419 and S799 strains (L_N_) was calculated and sorted in ascending order (Figure S10a). In the non‐biased simulation based on random MI segregation (Figure S9), equational segregation and non‐disjunction should occur at the same probability (Figure S10b). On the other hand, assuming that length‐dependent pairing occurs on some homologous chromosomes in meiosis I, the ratio of equational segregation to non‐disjunction should not be 50:50 (Figure S10c). We then defined the ratio of *X*_*N*_ (%) as the non‐disjunction probability of chromosome *N*. Using these *X*_*N*_, we calculated segregation probability of chromosome *N* (Figures 7e and S10d). In this simulation, *X*_max_ for the longest chromosome 4 was increased from 50% to 100% in 10% increments, while *X*_min_ for the smallest chromosome 1 was fixed at 50% in all cases. The segregation probability of the other chromosomes was estimated according to their lengths and are plotted in 10% increments as described in Section 4 (Figure S10d). Pa and Pb for every *X*_max_ were also recalculated (Figure S10e). Using these values, we simulated expected values of aneuploidy counts (Figure 7e), which exhibited very similar distribution with the observed results as *X*_max_ increased up to 100%.

## 3 DISCUSSION

### 3.1 Mechanisms for aneuploid formation in Spo11‐less meiosis

The whole genome sequencing results of the *spo11*∆ viable spores and the computer simulation analysis supported the notion that aneuploids are mainly formed in the random assortment of homologs during meiosis I. Sister chromatids are then distributed by equational segregation during meiosis II. These results mean that even in achiasmatic segregation during meiosis I of *spo11*∆ diploids, homologs consisting of a pair of sister chromatids retain unidirectional type centromeres that ensure reductional segregation.

Cells must have at least one set of all 16 chromosomes to produce viable spores after random segregation in meiosis I. In *spo11*∆, the probability of this event after the random segregation of homologs is expected to be extremely low, thereby *spo11*∆ can produce very few viable spores. It should be noted that all the spores carrying disomy inherited at least one chromosome from each parent and there were no spores with homozygous disomy (all disomic chromosomes are derived only from the single parent). These experimental results indicate that sister chromatids of homologs are accurately segregated to daughter cells during meiosis II in *spo11*∆, suggesting that centromeres of homologs in *spo11*∆ meiosis II take a bidirectional configuration.

### 3.2 Chromosomal variation of the aneuploid formation

In this study, we have demonstrated that the incidence of aneuploidy formation varies greatly among chromosomes during meiosis in the absence of Spo11. From the results, we consider that there are at least two factors determining the aneuploid incidence: the first is chromosome length. Indeed, chromosomes 2, 5, 6, 7, 9, 10, 11, 12, 13, and 14 showed relatively constant disomy formation per chromosome length (10–20 disomy formation per Mbp, Figure 4c). A linear approximation of aneuploid counts versus chromosome lengths among these chromosomes showed a high correlation (*R* = .899) (Figure 4e), suggesting that the aneuploid formation in Spo11‐deficient meiosis generally depends on chromosome length. This tendency was also seen in the simulation considering chromosome length‐dependence of MI homolog segregation (Figure 7e). Although longest chromosome 4 thought to be the most frequent aneuploid chromosome in the simulation, there was no aneuploid with chromosome 4. This discrepancy may be due to the survival bias with the effect of DSG impacts and the total gene number as described in below.

Why does aneuploidy more frequently occur in proportion to chromosome length during meiosis? In mitosis of human cancer cells, larger chromosomes showed decreased segregation fidelity which leads to a higher incidence of their incorporation into micronuclei (Bochtler et al., 2019). Therefore, it is plausible that longer yeast chromosomes also exhibit low segregation fidelity. On the other hand, Kumara et al. reported that longer chromosomes of diploid yeast tend to exhibit high chromosome loss rates during mitosis (Kumaran et al., 2013), which shows a stark contrast to the relationship between chromosome length and meiotic aneuploid frequency revealed in this study. We assume that the difference in chromosomal conditions in mitosis and meiosis may be a possible explanation for this discrepancy. In meiosis, it should be noted that the interaction between homologs in the budding yeast is partially observed even in the absence of Spo11 (Bhuiyan & Schmekel, 2004). In other words, it is presumed that homologs can behave in pairs to some extent even in Spo11‐less achiasmatic meiosis I. This would predict that meiosis I in *spo11∆* allows the movement of weakly interacting both parental homologs to the same pole at low frequency, rather than executing a completely random assortment of homologs. Since the interaction between homologs is thought to be stronger with longer chromosome lengths, aneuploidy may be more likely to occur in longer chromosomes during meiosis I.

Another possible factor is the total DSG impact on each chromosome. DSGs are genes sensitive to the excessive dosage which can prevent aneuploidy formation of their vehicle chromosome (Makanae et al., 2013). Multiple DSGs often reside on a chromosome and are predicted to synergistically suppress aneuploidy formation. Indeed, a logarithmic approximation of aneuploid incidence and the total DSG impact are inversely correlated (Figure 4f). The total DSG impact of chromosome 1 is remarkably low among all chromosomes (Figures 4f and S5), and this chromosome is exceptionally prone to aneuploidy. Chromosome 16 has the largest DSG impact, and this chromosome also has a low frequency of aneuploidy formation.

Chromosome 4 is the longest but did not cause any aneuploidy. Two major factors are possibly responsible for this result: the total number of genes associated with chromosome length and DSGs. First, chromosome 4 has the largest number of total genes. Torres et al reported that aneuploids with chromosome 4 aneuploidy exhibited a significantly slower growth rate than aneuploids with other chromosomes, and had a higher glucose requirement (Torres et al., 2007). Thus, such aneuploids require more glucose for mitotic transcription and protein translation than other aneuploids, leading to very low aneuploid tolerance. Second, the total DSG impact of this chromosome is moderate but has a very strong DSG gene *YDL192W* (*ARF1* encoding an ADP‐ribosylation factor, GTPase of the Ras superfamily involved in regulation of coated vesicle formation in intracellular trafficking within the Golgi apparatus) (Makanae et al., 2013), which allows only one additional copy.

However, there are a few chromosomes, such as chromosome 15, for which DSG effects or chromosome length alone cannot explain the frequency of aneuploidy formation. A previous study using the mitotic budding yeast demonstrated that aneuploid proliferation defects are not driven by copy number alterations of a few DSGs (Bonney et al., 2015). In addition, a study of aneuploid formation by conditionally inactivating CENP‐A in human cells revealed that inter‐chromosomal heterogeneity of centromeric status influences chromosome segregation fidelity (Dumont et al., 2020). Moreover, meiotic chromosomes take distinct features such as subnuclear compartments, centromere orientation, exchange of chromosomal proteins such as cohesins, and differences in mobility. These meiotic‐specific chromosome features may differently influence aneuploidy formation in specific chromosomes.

We also observed the bias of the partitioning of homologs from each parent. The frequency of YKN1419‐derived and S799‐derived homologs in *SPO11* ^+^ diploids was almost 50%, but *spo11*∆ viable spores exhibited a very slight bias toward S799‐derived homologs (Figure S6c,d,e). We attribute this phenomenon to the existence of a slight difference in surviving advantages of aneuploidy between homologs from both parents, which leads to the loss of aneuploidy during the second meiotic division and the nutritional divisions after spore germination.

### 3.3 Implication for human genetic diseases due to chromosomal abnormality

In this study, we found that the meiosis I‐derived aneuploid was selected in a chromosome‐specific manner. Human meiosis‐derived aneuploid also occurs in specific chromosomes, particularly in human chromosome aberration diseases such as Down syndrome and Klinefelter syndrome. In aneuploidy formation leading to human genetic disease, another issue must be considered, whether embryonic development can tolerate the presence of aneuploidy, which requires more multilayered considerations. This is probably the reason why the frequency of meiosis‐derived aneuploidy in humans is negatively correlated with chromosome length (Torres et al., 2008), a tendency apparently opposite to that observed in yeast meiosis. For instance, trisomy 21, which results in Down syndrome, is the only viable aneuploidy in humans, but trisomy 13 (Patau's syndrome) and trisomy 18 (Edwards' syndrome) die soon after birth. All three chromosomes are short and have fewer known coding genes than the other chromosomes (Torres et al., 2008). These features of the three chromosomes may result in a relatively high tolerance for aneuploidy formation compared with other chromosomes. Since yeast chromosomes are on average 1/170th shorter than human chromosomes, they have a much higher tolerance for aneuploid, and it is possible that a fundamental tendency not seen in human meiotic chromosomes could be more clearly recognized in this study.

We demonstrated that some chromosomes with meiosis I derived aneuploid such as chromosomes 2, 5, 7, 8, 10, 11, 12, 13, and 14 are not stable after the germination of the spores and are prone to be lost during multiple rounds of mitotic division. It has been reported that cells with aneuploid proliferate more slowly than euploid cells (Niwa et al., 2006; Niwa & Yanagida, 1985; Torres et al., 2007, 2008), possibly due to the protein stoichiometry imbalance of multi‐subunits proteins caused by altered gene dosage (Pavelka et al., 2010; Torres et al., 2007; Zhu et al., 2012). This leads us to consider that cells with such unstable aneuploids may be outcompeted by euploid‐type cells during multiple rounds of mitotic division. A previous study also indicated budding yeast aneuploid strains shed surplus chromosomes during mitotic passages and attempt to revert to euploid (Zhu et al., 2012).

It should be noted that a similar phenomenon called trisomy rescue (Lestou & Kalousek, 1998; Robinson et al., 1997) is observed in human fetus development. In genetic diseases with trisomy, the human embryo often eliminates cells with aneuploidy to restore the euploid karyotype (Greco et al., 2015), along with another mechanism called confined placental mosaicism (CPM), chromosomal differences between the fetus and placenta (Kalousek & Dill, 1983; Robinson et al., 1997). In one form of CPM, the mosaic cells with aneuploidy are often isolated to the trophectoderm, while the euploid cell line is isolated to the inner cell mass. These elaborate mechanisms of aneuploidy elimination during embryogenesis support human fetal development with normal karyotype.

### 3.4 Cancer development and chromosomal aneuploidy

A similar pathways of aneuploidy selection or exclusion is thought to function also in early human oncogenesis to prevent cancer development. Even in the budding yeast with very short chromosomes, DSGs have unneglectable impacts on the selection of a certain type of aneuploidy. Therefore, it is likely that putative human DSGs or similar genes may strongly drive the selection or exclusion of aneuploid in human cells. However, aneuploidy also functions to promote chromosome instability and genetic rearrangements. Aneuploidy has also been reported to contribute to cellular adaptation to stressful environments (Gilchrist & Stelkens, 2019; Torres et al., 2007; Zhu et al., 2012). Cancer may become malignant when the fitness of aneuploid cells is improved by being placed in a stressful environment of a tumor (e.g., low nutrition and hypoxia) or by additional genetic mutations that promote cell proliferation that overcome the low proliferative drawback of the aneuploid cells. Future research on the fundamental features of meiosis‐derived aneuploidy using yeast is expected to bring important insights into genetic diseases and cancer development.

## 4 EXPERIMENTAL PROCEDURES

### 4.1 Yeast strains and meiotic culture

Hybrid diploid strains of wild‐type YTG28 (*Mata/α LYS2/lys2 ho::LYS2/LYS2 leu2/leu2*(*asp718‐ecoRi) Arg4/arg4‐bgl CYH2/cyh2‐z*) and *spo11∆* YTG14 (*Mata/α LYS2/lys2 ho::LYS2/LYS2 leu2/leu2*(*asp718‐ecoRi*) *Arg4/arg4‐bgl CYH2/cyh2‐z spo11*∆*::URA3/spo11*∆*::URA3*) strains were used in this study. YTG28 was constructed by mating haploid parental strains with different backgrounds of YKN1419 (S288c‐derivative) and S799 (SK1‐derivative) with 0.7% of single nucleotide polymorphisms (SNPs). The *spo11*∆ haploid strains were constructed by replacing the *SPO11* locus with a *URA3* marker amplified with PCR primers (forward: 5′‐CATTAACTACTCTCCACCATACC, reverse: 5′‐TCCATTGAAGCTTAGTTTCTACC) for each haploid parental strain of YKN1419 and S799. YTG14 was generated by mating these *spo11*∆ parental haploid strains. The synchronous meiotic culture was performed basically as described previously (Fukuda et al., 2008; Ito et al., 2014; Kugou et al., 2009; Ohta et al., 1998). Strains were precultured in pre‐sporulation medium, SPS (0.5% yeast extract, 1% peptone, 0.17% yeast nitrogen base without ammonium sulfate and amino acids, 0.05 M potassium phthalate, 1% potassium acetate and 0.5% ammonium sulfate, pH 5.5), and shifted to sporulation medium (SPM, 1% potassium acetate). Cells were cultured at 30°C with vigorous aeration for 24 h.

### 4.2 Flow cytometric analysis

Cells after meiotic induction every hour from 0 to 8 h in SPM were fixed in 70% ethanol overnight followed by brief sonication. Cells were treated with 1 mg/ml RNase A in flow cytometry buffer (phosphate‐buffered saline (PBS), 137 mM NaCl, 2.7 mM KCl, 10 mM Na_2_HPO_4_, 1.76 mM KH_2_PO_4_, pH 7.4, containing 50 mM EDTA) over 4 h at 37°C then stained with 2.5 μg/ml propidium iodide (PI) in the flow cytometry buffer and analyzed by a NovoCyte flow cytometer (Agilent Technologies, USA).

### 4.3 Monitoring of meiotic progression by microscopic observation

Cells were fixed in 70% ethanol and stained with 1 μg/ml of 4,6‐diamidino‐2‐phenylindole (DAPI). The DAPI‐stained cells were then analyzed by fluorescent microscope BZ‐X810 (Keyence, Japan) to distinguish the fraction of bi‐nucleate (post‐MI) and tetra‐nucleate (MII) cells. The fraction of nuclei per one ascus was counted at 24 h of sporulation culture in SPM. Imaging analysis of the DAPI stained area was performed by Image J. Spore viability (mean + SD, *n* = 3) was estimated on YPD (10 g/L yeast extract, 20 g/L polypepton, 20 g/L glucose, 0.4 g/L adenine) plates after tetrad dissection. Exactly 334 wild‐type tetrad and 3137 *spo11*∆ tetrads were dissected and incubated at 30°C on YPD plates.

### 4.4 Viability estimation of return to growth assay

Return to growth (RTG) assay is an *S. cerevisiae*‐specific method on meiotic cells by shifting the cells from the sporulation process to the mitotic process (Esposito & Esposito, 1974). Meiotic cells in SPM are transferred to the mitotic growth medium (YPD plates), then returned to mitotic diploids. Cells cultured in the SPM medium for the indicated time (0–10 h) were transferred to YPD plates and the surviving colonies were counted.

### 4.5 DNA sample preparation and whole genome sequencing

Genomic DNA from wild‐type and *spo11*∆ viable spore progenies after the tetrad dissection were extracted using Dr.GenTLE™ (TAKARA Bio, Japan), according to the manufacturer's protocols. The DNA fragments were sheared to 300 bp using Covaris Focused‐ultrasonicator S220 (Covaris, USA), then sequencing gDNA libraries were prepared by using the NEBNext Ultra II DNA Library Prep Kit for Illumina (New England Biolabs, USA) or NEBNext Multiplex Oligos for Illumina (New England Biolabs, USA). Prepared gDNA libraries were subjected to the paired‐ended sequencing using HiSeq X (2 × 150 bp, Illumina, USA).

### 4.6 Re‐sequencing of the reference genomes of parental strains

The reference genomic sequence of SK1‐derived S799, one of the two parental haploids, was previously determined (Muramoto et al., 2018). The other parental strain YKN1419 is close to the standard laboratory strain S288C. S288C genome reference obtained from SGD (Saccharomyces Genome Database) (Cherry et al., 2012) was partially modified by using the HiSeq (illumine, USA) paired‐end sequencing data of YKN1419 in this study. Briefly, the HiSeq reads were mapped to the S288C genome reference using a Burrows–Wheeker Alinger (BWA) (Li & Durbin, 2009), and the SNPs and InDels were detected and modified by Genome Analysis ToolKit 4 (GATK4) (McKenna et al., 2010), VCFtools (vcftools_0.1.13) (Danecek et al., 2011) and YASS (Noé & Kucherov, 2005). The draft genome YKN1419 was corrected again by re‐mapping the HiSeq X data described above. The two haploid reference sequences were combined and used as diploid parental reference sequences to map and analyze the viable spore genomes.

### 4.7 Detection of recombination and genetic alterations of wild‐type and *spo11*∆ spores

The HiSeq reads of the *spo11*∆ viable spores were mapped to the above‐mentioned diploid parental reference sequences using BWA and called genetic variations using GATK4 HaplotypeCaller2 with the default parameters. The called variants were filtered with VCFfilter in vcftools (DP > 15, QD > 20, AF > 0.5, MQ > 30) and took the differences from the reference sequences using bcftools (Li, 2011). Ploidy of each *spo11*∆ viable spore was determined in two different ways, “SNP plots” and “coverage plots” as shown in (Figures 2 and 3). SNP plots were created by using SNP frequencies against each parental reference. Properly mapped paired reads with mapping quality >30 were filtered by SAMtools (Li et al., 2009) when mapped to diploid parental reference. Coverage information was smoothed by R function “SMA” and plotted per chromosome. The detailed plotting method of SNP plots is indicated in Figures 2 and 3. Crossing over (COs) and gene conversions (GCs) were detected by the RecombineX package (Li et al., 2022).

### 4.8 Mitotic passages of viable spores with aneuploids and analysis of aneuploid stability

The genomic DNA of the *spo11*∆ viable spores was isolated after mitotic growth of germinated spores for a few days. These samples were defined as g‐0 (generation 0). The g‐0 cells were further cultured for 110 generations in a liquid YPD medium. The doubling time was estimated as 2 h by the cell count of YKN1419 (haploid parental strain) in a liquid YPD medium. Frozen stock of g‐0 cells was plated on YPD plates and cultured overnight, then suspended in 5 ml of YPD liquid culture and cultured for a period corresponding to 7 generations (14 h). Subsequently, the cells were passaged twice a day (for periods corresponding to 4 or 7 generations) for 10 days until they were supposed to reach 110 generations. These cells were defined as g‐110 (generation 110). The whole genome sequences of the g‐0 and g‐110 cells were determined by HiSeq X. The sequence reads were mapped as described above. Next, the coverage differences between g‐110 and g‐0 disomy were analyzed. Since some disomic chromosomes were unstable and easy to be lost during mitotic culture, the average coverage of the g‐110 monosomy was normalized by the average coverage of their ancestor g‐0 monosomy, and each disomy was normalized by the average coverage of the monosomies in the same cell to calculate the rate of coverage change in disomy.

### 4.9 Simulation of aneuploid formation

We simulated probabilities of chromosome loss in each of 10^8^ *spo11*∆ spores having distinguishable two sets of 16 chromosomes. Then we estimated the duplicated chromosome numbers and calculated their viability. In our simulation, we made some assumptions according to the experimental data. More than one copy of all 16 chromosomes should exist for the survival of spores. We also assumed random and unequal chromosome segregation in meiosis I was caused by the lack of Spo11‐dependent meiotic recombination and equal segregation of sister chromatids in meiosis II. Certain chromosomes can be lost in the MII phase, which leads the two viable spores with identical chromosome sets (copies) to two viable spores with partial chromosome losses (altered copies) or one viable spore (single). We defined probabilities “a, (Pa)” and “b, (Pb)” that are the possibilities of monosomy and disomic chromosome loss in MII, respectively. Then we simulated meiotic chromosome segregation following the above assumptions. The resulting chromosome sets in each spore were checked whether they survived and kept disomy and their chromosome patterns were compared with calculate the ratio of identical or unbalanced twin and single spores. Simulation code is attached in the Data S2.

### 4.10 Refined simulation considering chromosome length‐dependence

In the simulation above, *spo11*∆ was assumed to show non‐biased random MI segregation, that is, ideally each of all homologous chromosome segregation patterns was expected to be 50% of equational segregation and 50% of non‐disjunction (Figure S10b). In the simulation considering chromosomal length‐dependence of MI segregation, homolog segregation is still random but biased according to the chromosome length. The average chromosome lengths of a certain chromosome *N* were calculated and described as *L*_*N*_ (*N* = 1, 2, …, 16). The minimum and maximum chromosome lengths are *L*_min_ and *L*_max_. Let $X_{N}$ be the probability of non‐disjunction occurrence of chromosome *N*. When the MI segregation is unbiased, in all chromosomes, the probability $X_{N}$ is

$$X_{N}=50\%.$$

Since the lack of Spo11 causes the randomness of homolog segregation, the minimum non‐disjunction occurrence ratio would be >50%. The minimum bias $X_{\min}$ for the shorted chromosome could be larger than 50%, but to set the model simple, $X_{\min}$ was always set to 50% in this simulation. When the maximum bias $X_{\max}$ for the longest chromosome, the probability of biased random segregation for chromosome *N* can be

$$X_{N}=\frac{L_{N}-L_{\min}\;}{L_{\max}-L_{\min}\;}\left(X_{\max}-X_{\min}\right)+X_{\min}.$$

We performed simulation of chromosome length dependently biased segregation when $X_{\max}$ was 50%, 60%, 70%, 80%, 90%, and 100%. The final proportions of the cell numbers of copies/(copies + altered copies), and the cell numbers of single/alive cells were used as constraints of searching the optimal Pa and Pb values.

## CONFLICT OF INTEREST

The authors declare no competing financial interests.

## Supporting information


**Data S1.** Supporting information figures.

**DATA S2.** Simulation code of java format.

## Data Availability

Sequencing data were deposited in the DDBJ database under BioProject accession number DRA014938. All other data are available from corresponding authors upon request.
