# Supplementary material for: Chromosome‐dependent aneuploid formation in Spo11‐less meiosis
Source: Genes Cells. 2023 Jan 5;28(2):129–48. doi: 10.1111/gtc.12998 (PMC10107155; doi:10.1111/gtc.12998)
Supplement: Supplementary file 1 — Data S1. Supporting information figures. [file GTC-28-129-s002.pdf]

Kawashima *et al.* Figure S1

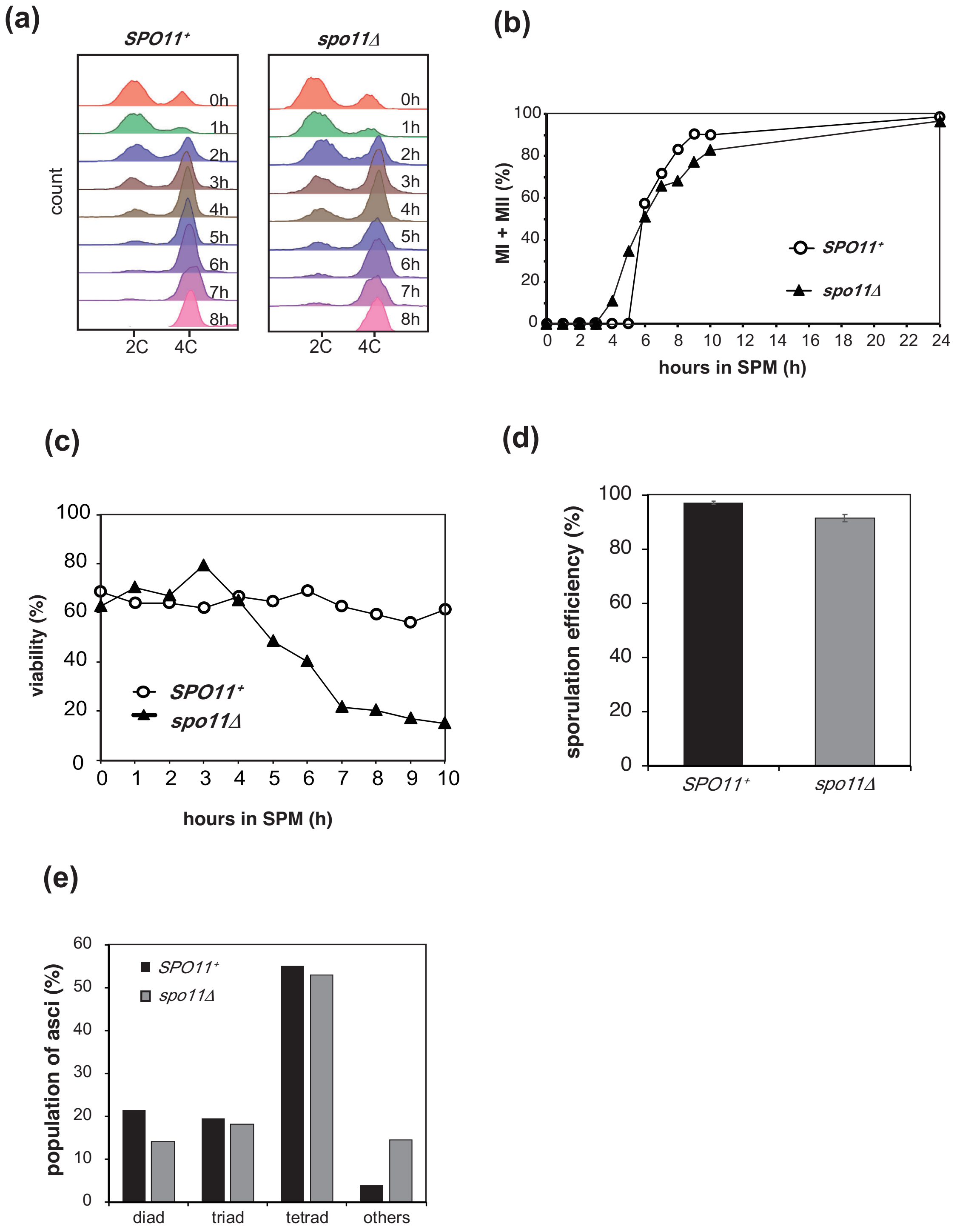

**Supplementary figure 1 Meiotic progression of the tester *SPO11*$^+$ and *spo11*Δ strains**

(a) Propidium iodide (PI) stained *SPO11*$^+$ and *spo11*Δ strains were analyzed by flow cytometry during meiosis (0-8 h). Flow cytometric histograms indicate the cell count, and 2C or 4C represent two or four copies of the chromosomal DNA. Both strains underwent synchronized meiosis at similar speeds.

(b) Meiotic divisions of DAPI-stained *SPO11*$^+$ and *spo11*Δ cells were analyzed by microscopic observation. The proportions of cells that completed meiosis I (MI) and meiosis II (MII) at indicated time points (MI + MII) are shown. More than 150 cells were counted at each time point. Open circles and filled triangles indicate *SPO11*$^+$ and *spo11*Δ, respectively.

(c) Viability of cells subjected to Return-to-Growth (RTG) experiments. Media were changed from sporulation medium SPM to nutritious medium YPD at each time point. Open circles and filled triangles indicate *SPO11*$^+$ and *spo11*Δ, respectively.

(d) Sporulation efficiency after 24 hours of meiosis (three biological replicates, mean + SD). Filled and grey bars represent results of *SPO11*$^+$ and *spo11*Δ, respectively.

(e) The number of spores with nuclei per ascus. DAPI-stained asci at 24 hours of meiosis were observed. Filled and grey bars represent results of *SPO11*$^+$ and *spo11*Δ, respectively.

Kawashima *et al.* Figure S2

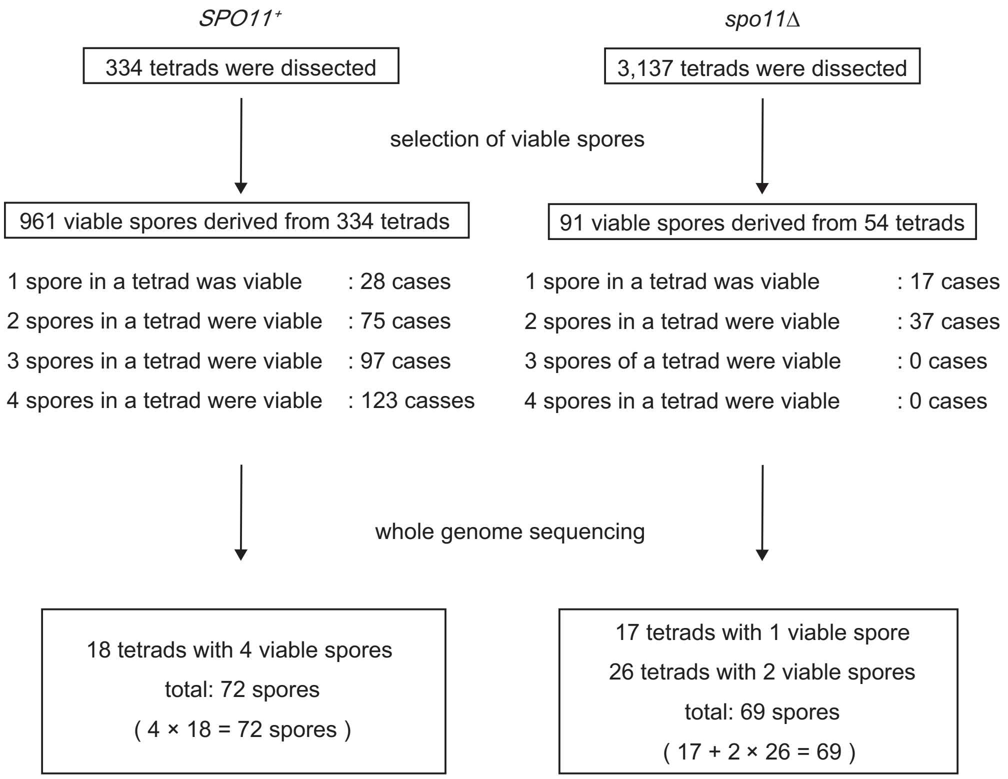

**Supplementary figure 2 Scheme of whole genome sequencing of dissected spores**

Schematic flowcharts of whole genome sequencing (WGS) of *SPO11*$^+$ and *spo11*Δ viable spores. We dissected 334 *SPO11*$^+$ tetrads and 3,137 *spo11*Δ tetrads. We analyzed 18 *SPO11*$^+$ tetrads that produced four viable spores as the control of the authentic meiosis with recombination. Of 3,137 *spo11*Δ tetrads, 54 *spo11*Δ tetrads produced viable spores. We found that 17 *spo11*Δ asci had only one viable spore, and 37 *spo11*Δ asci had two viable spores (2 x 37 = 74 spores), total 91 spores. We conducted WGS analysis of 72 *SPO11*$^+$ (4 spores in 18 asci) spores and 69 *spo11*Δ spores from single viable spores (17 spores) and two viable spores (2 spores in 26 tetrad; 52 spores).

# Kawashima *et al.* Figure S3

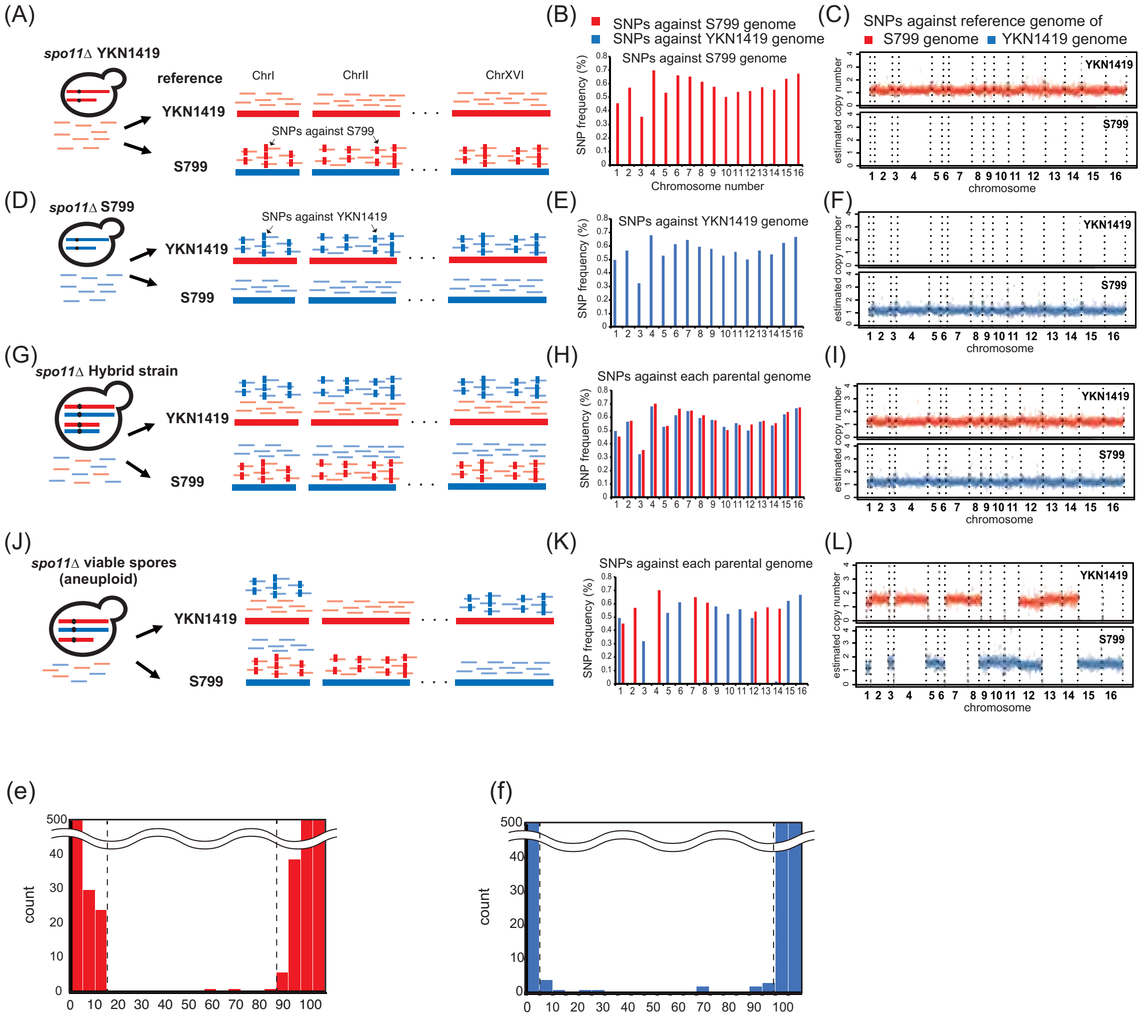


SNP frequency in *spo11Δ* viable spores against S799 reference genome, normalized by the number of SNPs in each chromosome

SNP frequency in *spo11Δ* viable spores against YKN1419 reference genome, normalized by the number of SNPs in each chromosome

**Supplementary figure 3 Schematic diagrams of aneuploidy and homolog identification**

(a-d) Procedures for the SNP-based discrimination of two parental homologs. The sequence reads were mapped to two parental haploid references, YKN1419 (red) and S799 (blue) genomes. When YKN1419-derived or S799-derived sequences were mapped with the S799 or YKN1419 references, respectively, SNPs were detected as indicated by filled squares in the left panels. Those SNP frequencies were normalized by chromosome length and plotted in the vertical bar plot (the middle panels, SNP plots). Red and blue bars show SNP frequencies against reference S799 and YKN1419 genomes, respectively. The right panels show the distribution of coverages of each chromosome mapped to parental references of YKN1419 and S799 (coverage plots). Red and blue dots represent mapped coverages to YKN1419 and S799 references, respectively.

(c) Sequence read mappings of a diploid hybrid strain. Both types of SNPs are detected at similar levels, leading to the appearance of red and blue bars at similar levels in the SNP plots (middle).

(d) An example of *spo11*Δ viable spores with aneuploidy. Some sequence reads are mapped to two parental references, the YKN1419 (red) and S799 (blue) genomes. In the SNP plot (middle), chromosomes 1 and 12 exhibit both red and blue bars, indicating that this spore has aneuploidy in chromosomes 1 and 12.

(e, f) Estimation of thresholds for SNP identification against the S799 and YKN1419 reference genome. Considering the presence of repetitive sequences, we determined the threshold values for SNP identification. The observed SNP frequencies in each chromosome among all *spo11*Δ spores compared to (e) S799 reference genomes and (f) YKN1419 reference genome normalized by the SNP frequencies of the wild-type (YKN1419 and S799

respectively) corresponding chromosome. When this value is close to 0% or 100%, the read is more identical to the reference genome sequences or the other parental genome sequence, respectively. We defined a S799-derived chromosome when SNP frequencies were lower than 15% and a YKN1419-derived chromosome when SNP frequencies were higher than 85% in (e). And We defined a YKN1419-derived chromosome when SNP frequencies were lower than 5% and a S799-derived chromosome when SNP frequencies were higher than 95% in (f).

**(a)**

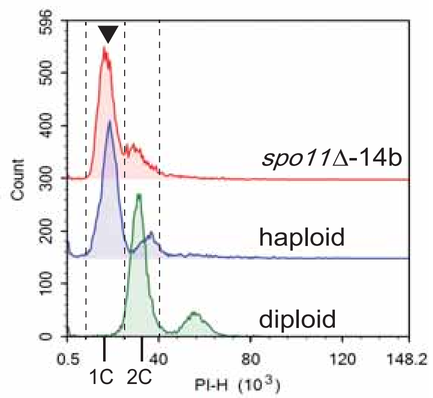


**(b)**

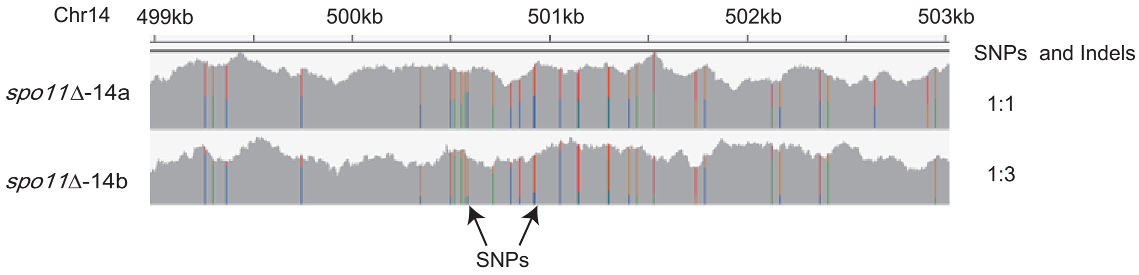


**Supplemental Figure 4 Altered copy number of aneuploids in *spo11*Δ-14b strain**

(a) Flow cytometric analyses of propidium iodide (PI) stained *spo11*Δ-14b cells in comparison with a haploid strain YKN1419 and a diploid strain (hybrid of YKN1419 and S799). Note that there is a shoulder between 1C and 2C peaks (shown with an arrowhead).

(b) Detection of SNPs within the 499 kb – 503 kb region of chromosome 14 in *spo11*Δ-14a (upper panel) and *spo11*Δ-14b (lower panel). Positions of SNPs are indicated with different colors (A, green; T, red; G, yellow, C, blue). Ratio of SNPs in each mapped read is 1:1 in *spo11*Δ-14a (upper panel), but is 1:3 or 3:1 in *spo11*Δ-14b (lower panel).

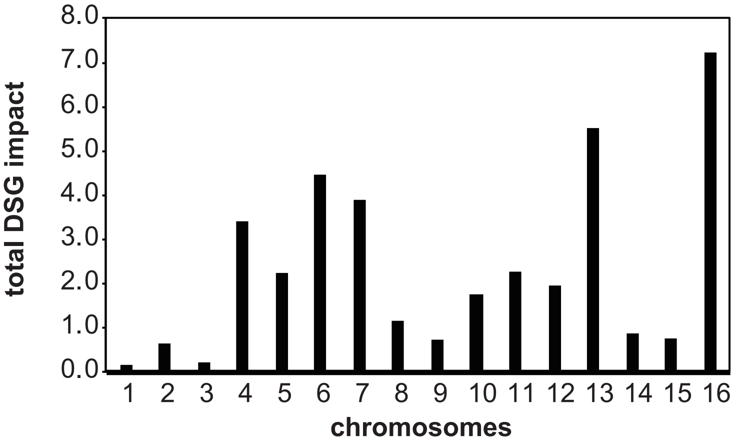


**Supplemental Figure 5 Total DSG impact of each chromosome**

The chromosomal tolerance for DSG for each chromosome. Vertical axis shows the total DSG impact of the chromosomes. The inverse of the copy number limit for each DSG was calculated and summed for each chromosome, which was defined as the total DSG Impact.

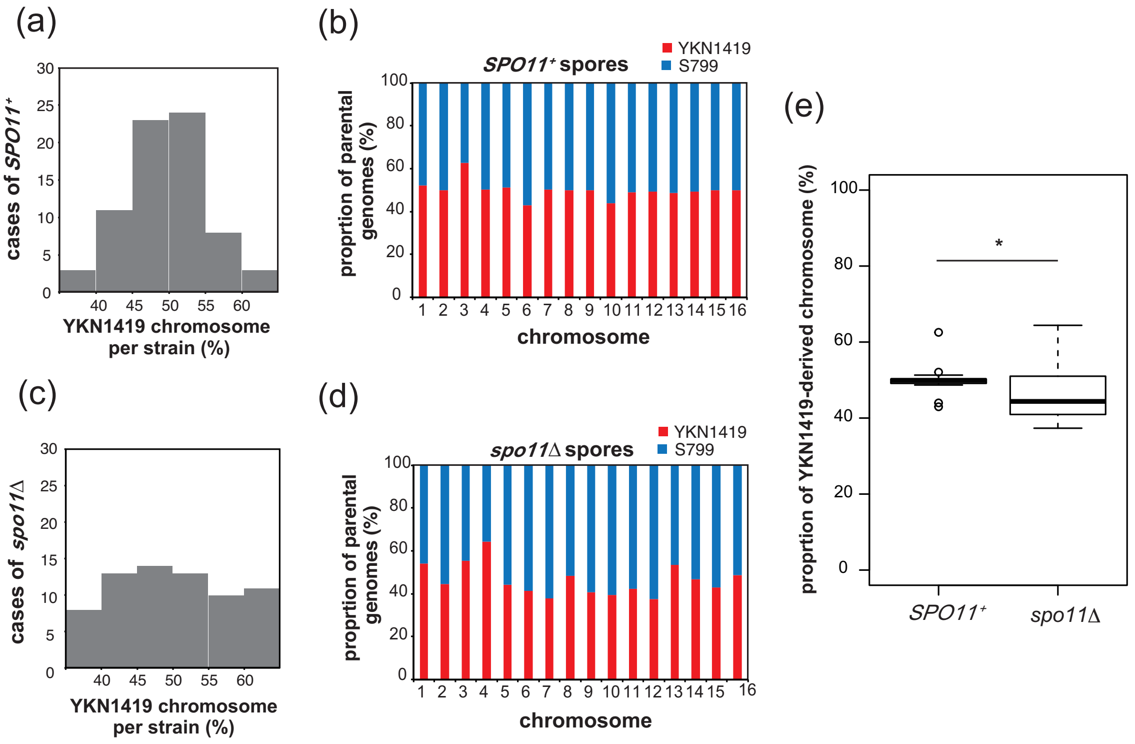


**Supplemental Figure 6 Imbalanced segregation of S799 and YKN1419 homologs during *spo11*Δ meiosis**

(a): Frequency of YKN1419-derived chromosomes in *SPO11*$^{+}$ spores

(b): Average frequencies of YKN1419 (red)- and S799 (blue)-derived sequences for each chromosome of *SPO11*$^{+}$ spores. Red and blue bars indicate YKN1419 and S799 sequences, respectively.

(c): Frequency of YKN1419-derived chromosomes in *spo11*Δ spores.

(d): Average frequencies of YKN1419 (red)- and S799 (blue)-derived sequences for each chromosome of *spo11*Δ.

(e): Frequencies of YKN1419-derived sequences in *SPO11*$^{+}$ and *spo11*Δ spores. Paired t-test was performed (*$p < 0.05$).

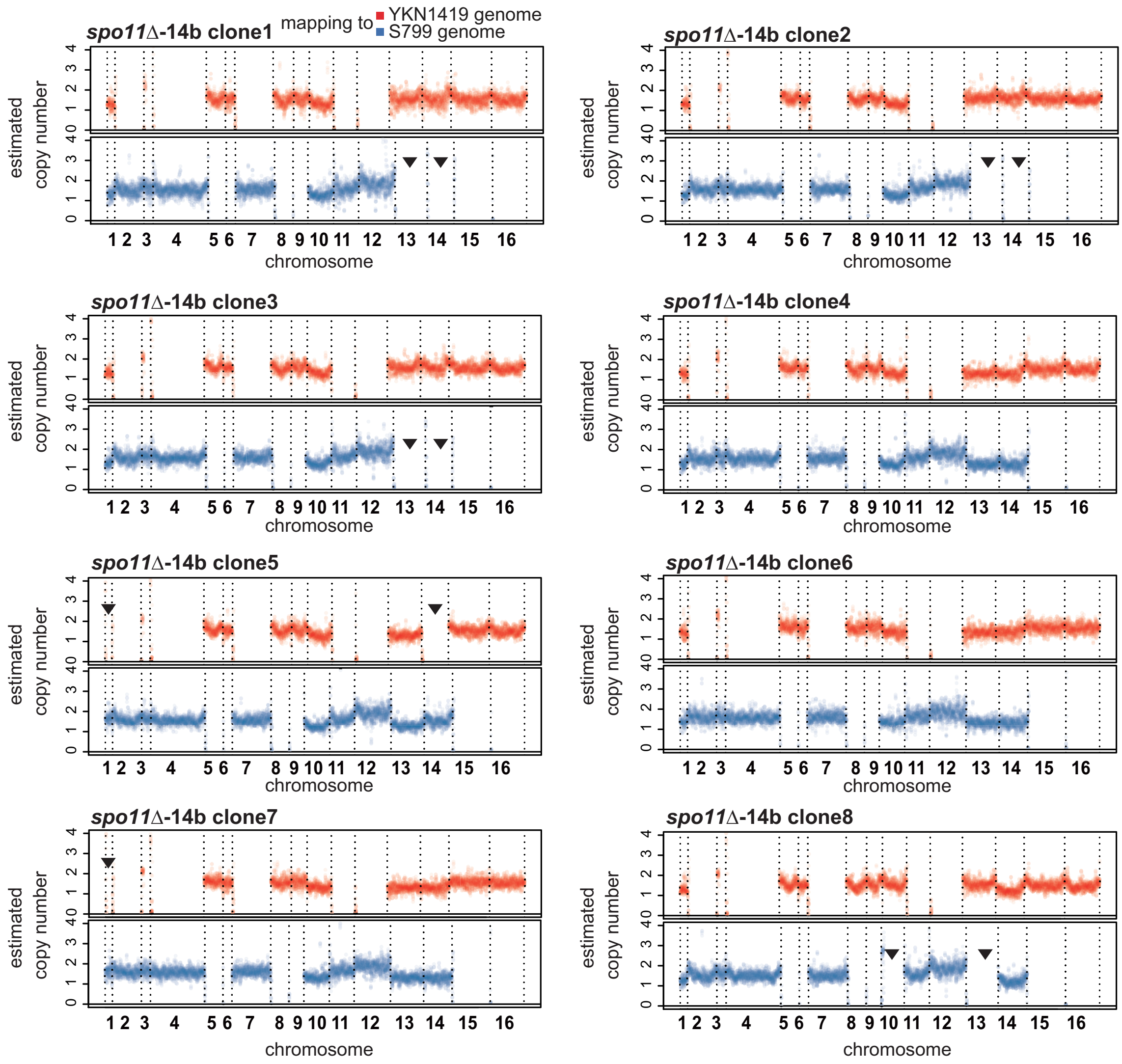


**Supplemental figure 7 Mappings of WGS data of 8 isolated progenies of *spo11*Δ-14b**

The distribution of coverages of each chromosome of 8 isolated progenies of *spo11*Δ-14 mapped to parental references of YKN1419 and S799. Red and blue dots represent mapped coverages to YKN1419 and S799 references, respectively.

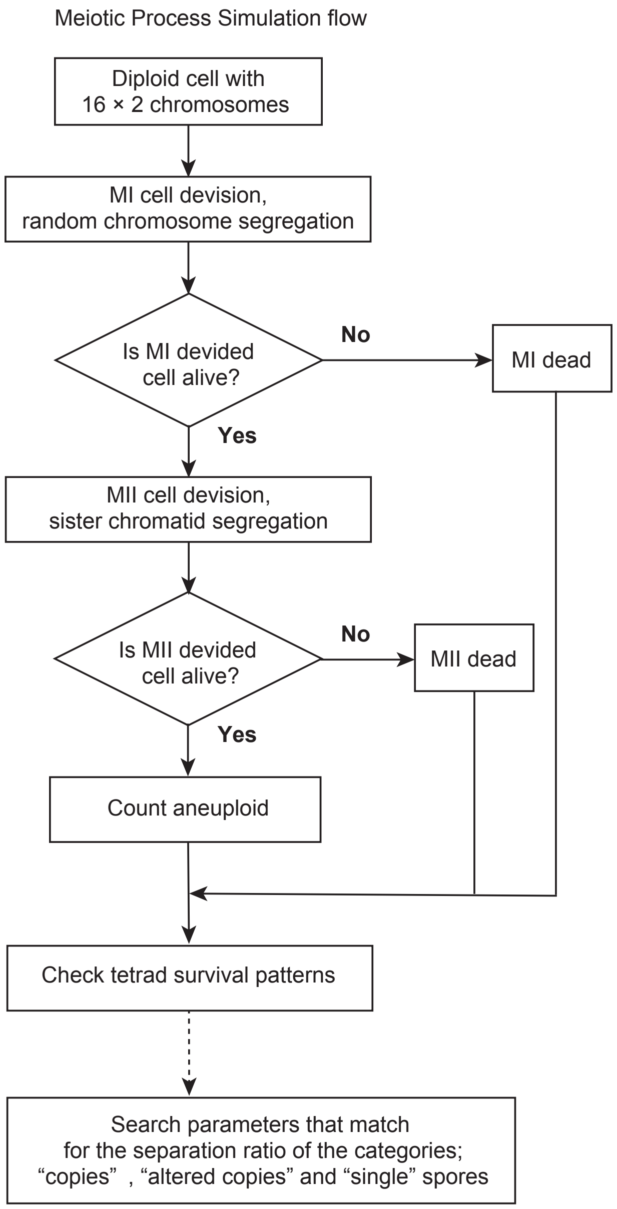


**Supplementary figure 8 Schematic flowchart of simulation of *spo11*Δ meiotic segregation**

Overview of searching the chromosome loss probability a and b, counting aneuploid and checking spore formation patterns.

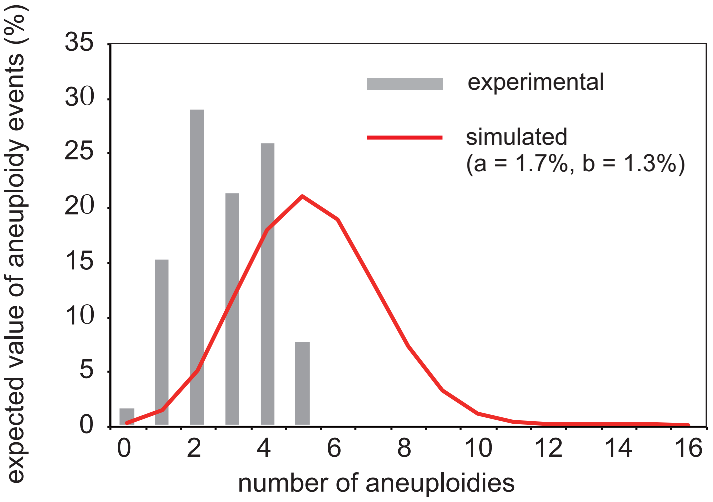


**Supplementary figure 9　Distribution of numbers of aneuploidy in *spo11*Δ spores.**

Grey bars indicate observed results. Red line indicates simulated results at Pa = 1.7% and Pb = 1.3%. The complete diploid spores were not included in this graph.

Kawashima *et al.* Figure S10

(a)

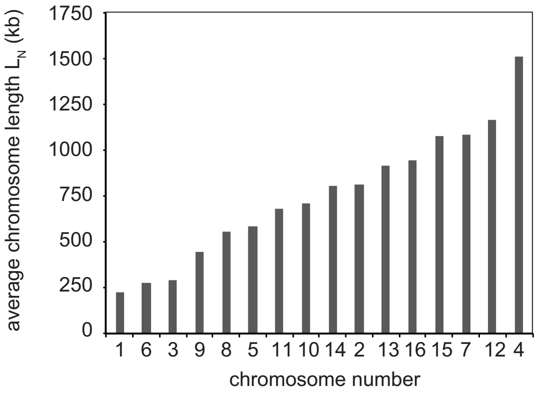


(b)

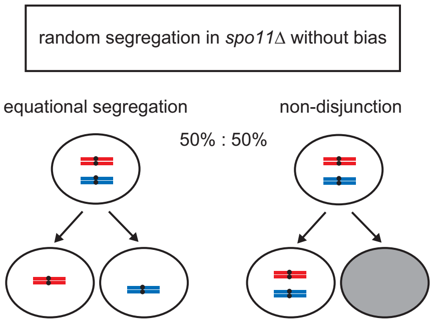


(c)

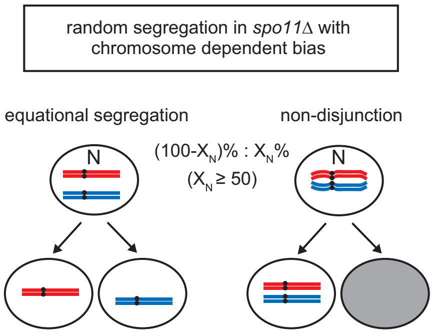


(d)

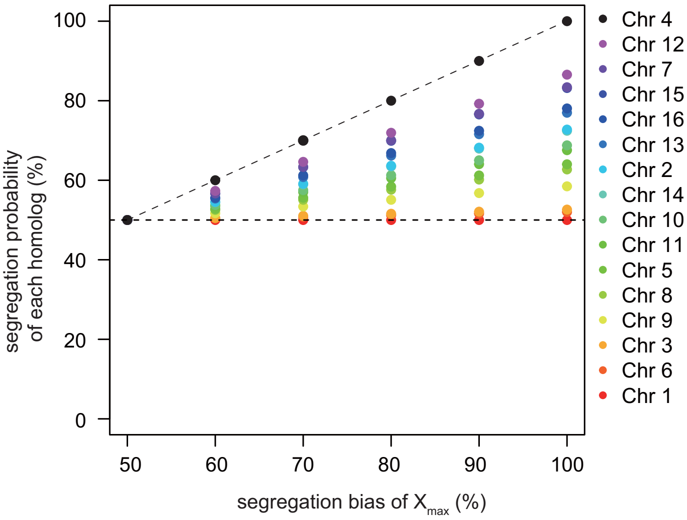


(e)

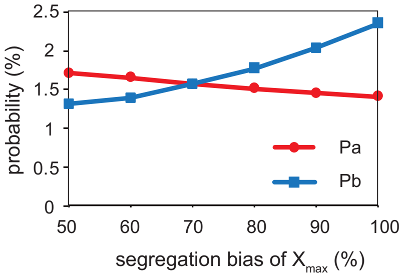

**Supplementary figure 10 Simulation of chromosome length dependent biased MI segregation**

(a) Distribution of chromosome length by ascending-order. Horizontal axis indicates the chromosome number. Vertical axis indicates the average chromosome length of YKN1419 derived and S799 derived homologs.

(b) Schematic diagram of random segregation in *spo11*Δ without bias. There are two segregation patterns for each homolog: equational segregation and non-disjunction. The probability of each pattern is 50% mutually. Even in the biased simulation, the segregation probability of the shortest chromosome was always set to this 50%:50% random segregation.

(c) Schematic diagram of random segregation of in *spo11*Δ with chromosome dependent bias. When the probability of non-disjunction occurrence of chromosome N is defined $X_N$ %, the probability of equational segregation is (100 - $X_N$) %. In the biased simulation, the values of $X_N$ were changed ($50 < X_N \leq 100$).

(d) Distribution of segregation probabilities with bias proportional to the chromosome length in the biased simulation. Each rainbow-colored dot in a row shows the segregation probability of each homolog in a simulation trial. Horizontal axis indicates the segregation bias of $X_{max}$ from 50% to 100%. When $X_{max}$ is 50%, it means all $X_N$ values are 50% and non-biased segregation is expected as described in (b). When $X_{max}$ is larger than 50%, segregation probabilities for each chromosome are linearly biased depending on their chromosome length as described in (c) and the Experimental Procedures section.

(e) Transition of Pa and Pb, depending on various MI segregation bias. The final ratio of the cell numbers of copies/ (copies + altered copies), and the cell numbers of single/ survived cells were fixed to the experimentally observed values, and the optimal Pa and Pb were searched by simulations. Red and blue lines indicate Pa and Pb, respectively.
